# Enteric-coating film effect on the delayed drug release of pantoprazole gastro-resistant generic tablets

**DOI:** 10.12688/f1000research.140607.1

**Published:** 2023-10-12

**Authors:** Mosab Arafat, Molham Sakkal, Mohammad F. Bostanudin, Othman Abdulrahim Alhanbali, Priya Yuvaraju, Rami Beiram, Bassem Sadek, Amal Akour, Salahdein AbuRuz

**Affiliations:** 1 College of Pharmacy, Al Ain University, Al Ain, Abu Dhabi, 64141, United Arab Emirates; 2 Department of Pharmacy, Faculty of Medicine and Health Sciences, An-Najah National University, Nablus, Palestinian Territory; 3 Department of Pharmacology and Therapeutics, College of Medicine and Health Sciences, United Arab Emirates University, Al Ain, Abu Dhabi, 17666, United Arab Emirates; 4 Department of Biopharmaceutics and Clinical Pharmacy, School of Pharmacy, The University of Jordan, Amman, Amman Governorate, 11942, Jordan

**Keywords:** Enteric Coating Film; Pantoprazole; In vitro Drug Release; Analytical Techniques; Differential Scanning Calorimetry; Thermogravimetric Analysis; Generic Drug; Polysorbate 80.

## Abstract

**Background:** Enteric coating films in acidic labile tablets protect the drug molecule from the acidic environment of the stomach. However, variations in the excipients used in the coating formulation may affect their ability to provide adequate protection. This study is the first to investigate the potential effects of coating materials on the protective functionality of enteric coating films for pantoprazole (PNZ) generic tablets after their recall from the market.

**Methods:** A comparative analysis was conducted between generic and branded PNZ products, using pure drug powder for identification. The *in vitro* release of the drug was evaluated in different pH media. The study also utilized various analytical and thermal techniques, including differential scanning calorimetry (DSC), thermogravimetric analysis (TGA), X-ray diffraction (XRD), scanning electron microscopy (SEM), Fourier-transform infrared (FTIR), and confocal Raman microscopy.

**Results:** The *in vitro* assessment results revealed significant variations in the release profile for the generic product in acidic media at 120 min. DSC and TGA thermal profile analyses showed slight variation between the two products. XRD analysis exhibited a noticeable difference in peak intensity for the generic sample, while SEM revealed smaller particle sizes in the generic product. The obtained spectra profile for the generic product displayed significant variation in peaks and band intensity, possibly due to impurities. These findings suggest that the excipients used in the enteric coating film of the generic product may have affected its protective functionality, leading to premature drug release in acidic media. Additionally, the presence of polysorbate 80 (P-80) in the brand product might improve the properties of the enteric coating film due to its multi-functionality.

**Conclusions**: In conclusion, the excipients used in the brand product demonstrated superior functionality in effectively protecting the drug molecule from acidic media through the enteric coating film, as compared to the generic version.

## Introduction

Enteric coating films play a crucial role in pharmaceutical formulations as they are specifically designed to provide protection from premature releases of the drug molecule in acidic media. ^1^^,^^2^ This protective function is particularly essential for drugs that are susceptible to degradation in acidic conditions, such as erythromycin, ^3^ ampicillin, ^4^ and penicillin G antibiotics, ^5^ as well as certain proton pump inhibitors class of drugs, including omeprazole, ^4^ Pantoprazole sodium sesquihydrate (PNZ), ^6^ and esomeprazole. ^5^^,^^7^ By forming a protective barrier, enteric coating films ensure the drugs reach their intended site of action intact. ^5^^,^^7^ Moreover, enteric coating films also serve to prevent local irritation of the stomach mucosa caused by certain acidic drugs, ^8^^,^^9^ including NSAIDs, like diclofenac ^10^ and valproic acid. ^9^ This feature is particularly important for enhancing patient tolerance and reducing potential side effects. ^8^^–^^10^

Enteric-coated properties for tablets are commonly achieved through the use of various polymers and additives. ^11^^–^^13^ Anionic polymers containing carboxyl groups are frequently employed to achieve the desired enteric effects. ^14^ These polymers are insoluble under low pH conditions but exhibit solubility in intestinal fluid as the pH increases due to the ionization of acidic functional groups, resulting in polymer swelling. ^11^^,^^14^ Common examples of anionic polymers used for enteric coating include cellulose acetate, ^15^ polyvinyl acetate, ^16^ hydroxypropyl methylcellulose, ^17^^,^^18^ and methacrylic acid copolymers. ^11^^–^^13^ Additives play a crucial role in polymer formulations, enhancing mechanical properties, ^19^ modifying film permeability, ^19^ facilitating film formation, ^12^ and improving processing. ^12^^,^^20^ Several commonly used additives serve these purposes effectively. For instance, plasticizers like Polysorbate 80 (P-80), ^21^ tributyl citrate, ^22^ and diethyl phthalate ^23^ increase film flexibility, reduce brittleness, and influence drug release by minimizing crack formation. ^12^^,^^24^ Anti-adherent materials, such as Talc ^25^^,^^26^ and Glyceryl monostearate, ^27^ are employed to reduce film tackiness and prevent substrate agglomeration. ^12^^,^^28^ Additionally, surfactants like P-80, ^29^ sorbitan monooleate, ^30^ and sodium dodecyl sulfate ^31^ are utilized to emulsify water-insoluble plasticizers, improve substrate wettability, and stabilize suspensions, thereby enhancing the overall properties of the enteric coat film. ^12^^,^^29^

Generic drugs are manufactured by pharmaceutical companies in accordance with FDA. ^32^^,^^33^ These regulations mandate that generic drugs have the same active ingredients, dosage form, strength, and route of administration as their brand-name counterparts and must be bioequivalent. ^26^^,^^32^^,^^34^ However, certain variations in excipients are permitted. ^35^^,^^36^ Although generic drugs are generally considered bioequivalent, some enteric-coated generic drugs have shown compromised functionality in protecting the drug molecule, resulting in premature drug release in simulated acidic media at physiological pH, ^37^^–^^39^ such as omeprazole enteric-coated capsules, ^39^ diclofenac enteric-coated tablets, ^38^ lansoprazole enteric-coated tablets, ^40^ and PNZ enteric-coated tablets. ^37^

In this study, PNZ enteric-coated tablets were chosen as the model generic drug for comparison with the branded one, which was used as a reference. The generic product of PNZ was selected due to the reported issues that led to its recall from the local market. The chemical name of PNZ is Sodium 5-(difluoromethoxy)-2-(3,4-dimethoxy-2-pyridinyl)methyl)sulfincyl]-1H-benzimidazole sesquihydrate. ^41^ It is primarily used as an anti-ulcer agent to treat duodenal and gastric ulcers. ^11^^,^^42^ PNZ is classified as a class III drug according to the Biopharmaceutics Classification System (BCS), which indicates high solubility and low permeability. ^43^^,^^44^ PNZ has a molecular weight of 432.4 g/mol ^11^ and a melting point of 149-150°C. ^11^^,^^45^ It is highly soluble in water, ^46^ slightly soluble in chloroform, ^11^ and practically insoluble in n-hexane. ^11^ PNZ has a pKa value of 3.55 and a LogP value of 2.11. ^47^ PNZ is a drug that is susceptible to degradation in the acidic environment of the stomach. ^48^ therefore, commercially available enteric-coated tablets or capsules of PNZ are used to avoid the drug molecule degradation in acidic environments, ensuring its effectiveness. ^37^^,^^49^

Several research studies have reported a reduction in the function of the enteric coating film, attributed to multiple possible factors. ^2^^,^^50^^–^^52^ Therefore, maintaining the integrity of the enteric coating film primarily relies on the type and quantity of polymers and additives used for the film coating, as well as the manufacturing process. ^2^^,^^53^ Several research studies have emphasized the importance of analyzing and evaluating the quality and functionality of enteric-coated medications using a range of analytical and thermal techniques. ^37^^,^^39^^,^^45^ For instance sesquihydrate PNZ generic medication showed a different thermal profile than monohydrate PNZ when subjected to differential scanning calorimetry (DSC) and thermal gravimetric analysis (TGA). ^45^ Fourier transforms infrared (FTIR) and Raman spectroscopy also revealed a significant difference in the spectra between monohydrate and sesquihydrate forms of PNZ drug molecule. ^45^ In another study, premature drug release from enteric-coated generic PNZ tablets was observed in acidic media during *in vitro* dissolution evaluation. ^37^ Furthermore, scanning electron microscopy (SEM) and X-ray diffraction (XRD) examinations demonstrated the absence of enteric coating on some granules of generic omeprazole capsules. ^39^ These findings emphasize the importance of selecting suitable type and amount of ingredients to formulate the enteric coating film, as well as implementing an appropriate manufacturing process.

Therefore, the aim of this study was to find out the effect of coating film materials on the protection of PNZ drug molecules in acidic pH media. Both the brand and generic products of PNZ were utilized to compare any possible differences, with the brand product serving as the reference. Hence, the pure powder of PNZ was used for the drug identification process. To achieve these objectives, a range of analytical and thermal techniques, including DSC, TGA, XRD, SEM, FTIR, and Confocal Raman Microscopy, were used. Additionally, the *in vitro* drug release rate was evaluated in different pH media. To the best of our knowledge, no prior studies have been conducted on generic PNZ after has been recalled from the local market.

## Methods

### Materials

The generic product of PNZ and the brand product of PNZ were ordered from Boots Pharmacy (Dubai, UAE). The pure powder of PNZ was purchased from Sigma Aldrich (St. Louis, MO, USA).

### Tablets disintegration

The disintegration characteristics for both branded and generic tablets of PNZ were evaluated using the fully automated disintegration instrument (PTZ Auto EZ, Hainburg, Germany). The procedure was performed using 0.1 N hydrochloric acid as the disintegration medium for a duration of 1 h. The temperature was maintained at 37 ± 0.5°C and the specific disintegration time of each tablet was recorded. The expectation was that all six tablets from each product would disintegrate within the predetermined time frame.

### *In vitro* dissolution test

The drug release of branded and generic products of PNZ was evaluated using the Dissolution Apparatus 2 model Dis 8000 (Copley Scientific, Nottingham, UK) under controlled conditions. The dissolution was carried out at a constant temperature of 37 ± 0.5 ^0^C and a stirring speed of 75 rpm. During the incubation process, three different media with a volume of 900 mL were used, each with a pH value of 1.2, 5, and 6.8. At two time intervals, 60 and 120 min, a sample of 5 mL was taken from each vessel and replaced with an equal volume of distilled water. All collected samples were filtered to eliminate any unwanted particulate matter and diluted with distilled water. The drug concentration in each sample was measured through ultraviolet spectrophotometry analysis at a wavelength of 290 nm. To ensure the accuracy and reliability of the results, each product involved six samples (n=6).

### Differential scanning calorimeter (DSC)

DSC-60 Plus instrument (Shimadzu, Kyoto, Japan) was utilized to analyze the thermal profile for the pure powder of PNZ, the brand product of PNZ, and the generic product of PNZ. a precise amount of 3-5 mg powder was weighed and transferred into sample pans for each product. The analysis was conducted under controlled conditions, where the samples were scanned over a temperature range of 25-350°C at a rate of 10°C per min while being exposed to a continuous flow of nitrogen at a rate of 100 mL/min. The resulting data was collected and processed using the Lab Solutions TA software. To ensure the statistical significance of the results, the experiment was repeated six times for each product, resulting in a total of six samples (n=6).

### Thermogravimetric analysis (TGA)

The Thermal Gravimetric Analysis (TGA) of the three samples was carried out using the TGA-50 instrument (Shimadzu, Kyoto, Japan). For each sample, an accurate amount of 10-15 mg powder was weighed and loaded into an alumina pan for analysis. The analysis was performed by scanning the samples over a temperature range of 0°C to 600°C at a rate of 15°C per min, while being exposed to a continuous flow of nitrogen at a rate of 50 mL per min. The Lab solutions TA Thermal Analysis Workstation software was used to closely monitor and regulate the analysis process. To ensure the statistical significance of the results, the experiment was repeated six times for each product, resulting in a total of six samples (n=6).

### X-ray diffraction

The crystalline structure for the pure powder of PNZ, the brand product of PNZ, and the generic product of PNZ were determined using XRD 6100 (Shimadzu, Kyoto, Japan). XRD patterns were collected for the tablets and a reference standard by scanning 2θ over a range of 10° to 80° at a rate of 2° per min.

### Scanning electron microscopy

The JSM-6010PLUS/LA scanning electron microscope (JEOL, Tokyo, Japan) was used to examine the morphological characteristics for the pure powder of PNZ, the brand product of PNZ, and the generic product of PNZ. To prepare the samples for examination, a small amount was affixed to the specimen holder stub via double-coated adhesive carbon tape. Prior to the 20-kilovolt test, the sample was coated with a layer of gold for 10 min in a vacuum environment using the Cressington sputter coater 108 autos. After applying the gold coat, the sample was positioned on the sample stage and examined using the SEM In Touch Scope software version 2. The process was carried out with precision to ensure the quality of the analysis.

### Fourier- Transform infrared spectroscopy

The Fourier Transform Infrared spectra for the three samples were recorded using a Thermo Nicolet Nexus 670 spectrometer (GMI, Ramsey, USA). To facilitate the measurement process, each sample was separately mixed with dry Potassium Bromide at a ratio of 1:100 and subsequently compressed into pellets. The transmittance of the samples was measured within a range of 4000 cm ^-1^ to 450 cm ^-1^, with the spectra obtained from 32 scans. To ensure the accuracy and reliability of the data, the results were then processed using the OMNIC 9 software.

### Confocal microscope Raman spectroscopy

The Raman spectra of the pure powder of PNZ, the brand product of PNZ, and the generic product of PNZ were obtained using the Confocal Microscope Raman/PL System (NOST, Daejeon, Korea). The samples were placed on a glass slide and subjected to a 5s laser exposure using a 20X objective lens to perform Raman mapping. The Raman shift range of each sample was scanned, spanning from 0 cm ^-1^ to 4000 cm ^-1^, and the corresponding counts were recorded. The RAON-SPEC program was then utilized to analyze the obtained samples.

### Statistical analysis

The mean values of the determined variables were compared using the Independent Sample T-test for statistical analysis of variance. If *p < 0.05*, the differences were considered significant. The Statistical Package for Social Science (SPSS) Version 26 was used for the analysis.

## Results

### *In vitro* dissolution drug release and disintegration assessments

**Figure 1.**
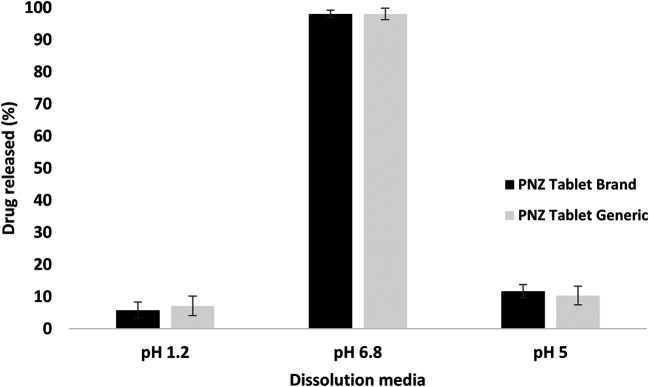
Comparison of dissolution profiles for the brand and generic products of Pantoprazole (PNZ in three dissolution media (pH 1.2, pH 5, and pH 6.8) after 60 min at 37°C. Values are expressed as means ± S.D. (n=6).

**Figure 2.**
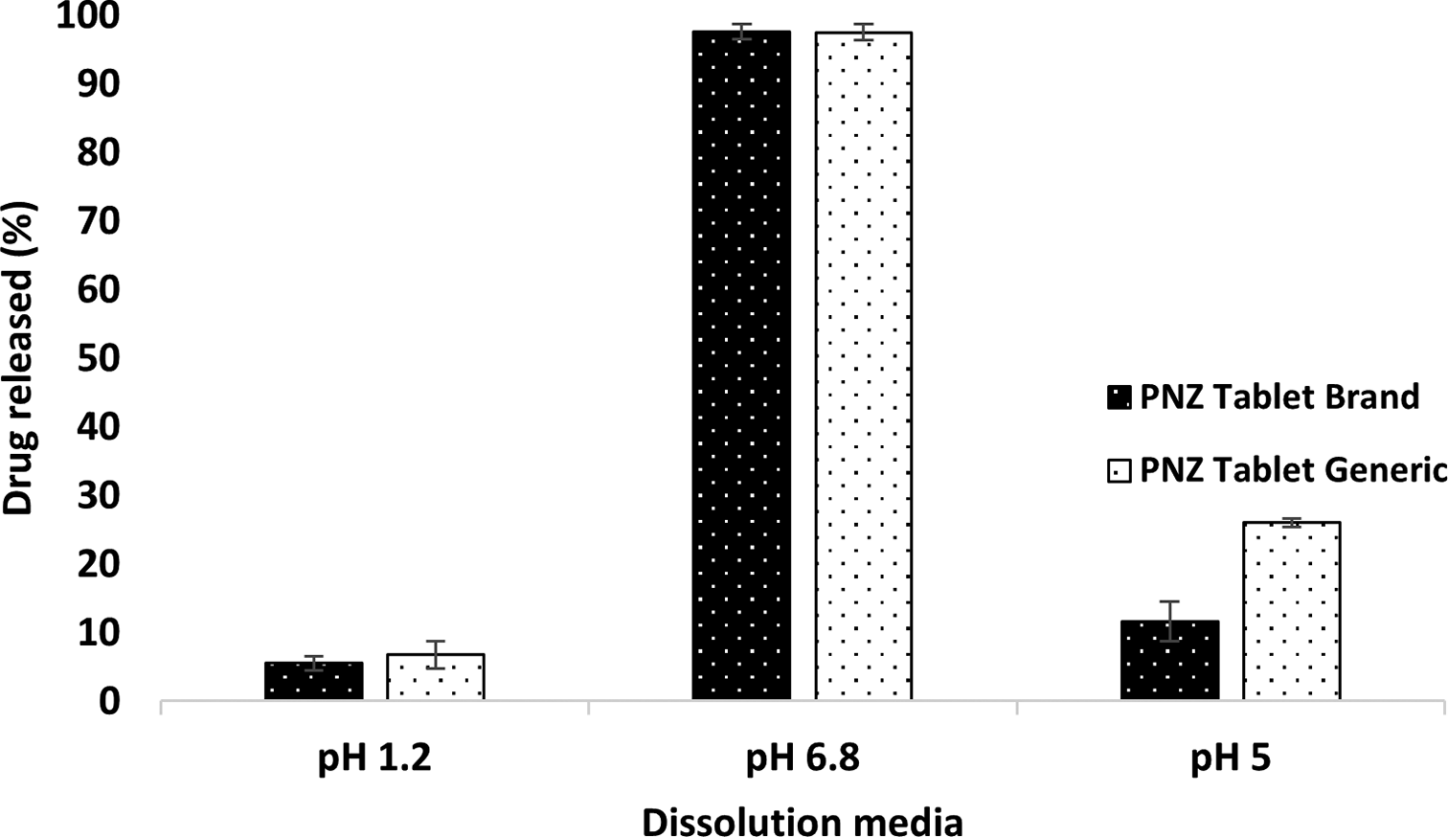
Comparison of dissolution profiles for the brand and generic products of PNZ in three dissolution media (pH 1.2, pH 5, and pH 6.8) after 120 min at 37°C. Values are expressed as means ± S.D. (n=6).

The drug released from the branded and generic product of PNZ exhibited only slight differences ( *p > 0.05*) in the first hour in the three different pH dissolution media, as presented in Figure 1. ^78^ However, Figure 2 revealed a significant difference ( *p < 0.05*) in the percentage of drug released between the two products in the pH 5 media after 120 min. The drug release exceeded 10% in the acidic media, which was not in compliance with the United States Pharmacopeia (USP) specifications for enteric-coated tablets. ^37^ In contrast, the brand product PNZ complied with the USP specifications for drug release in acidic media. ^37^ These findings were further supported by the disintegration results, whereas the generic tablet disintegrated in the acidic media, while the branded tablet demonstrated resistance to the same media.

### Differential scanning calorimetry

**Figure 3.**
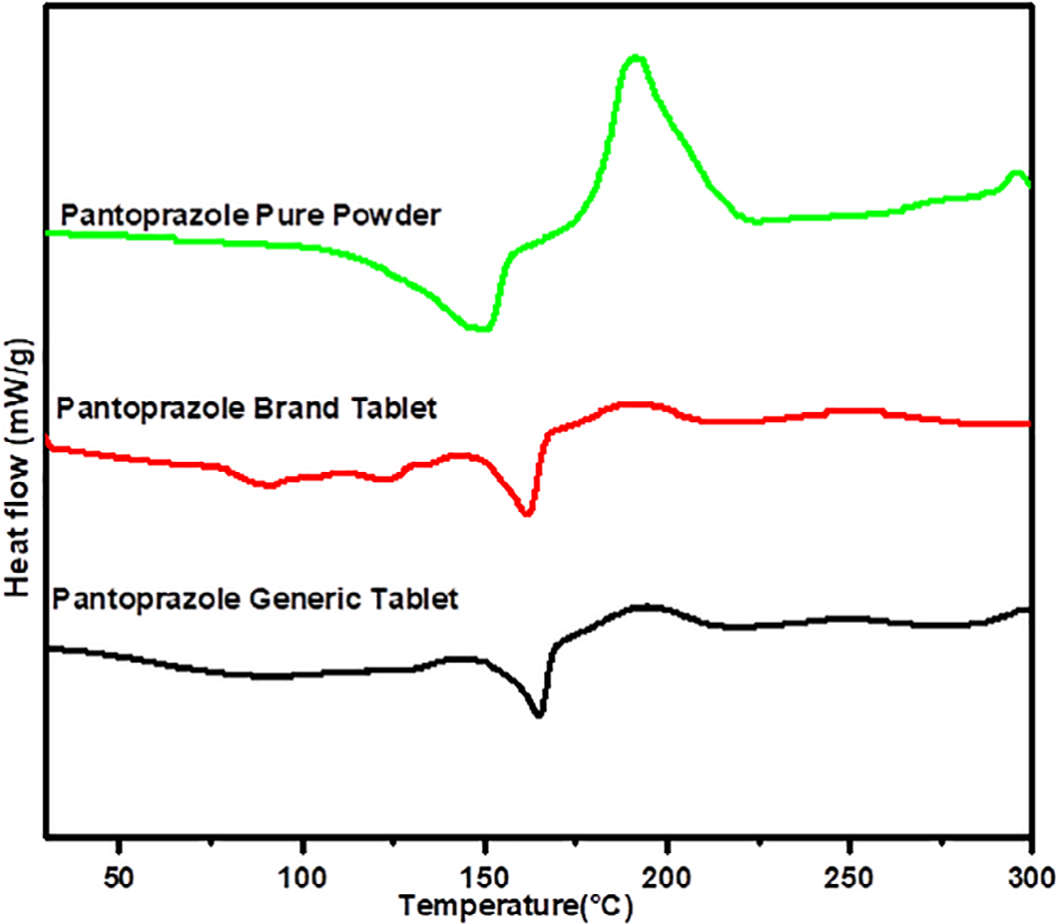
DSC Thermal analytical spectra of the pure powder of PNZ, the brand product of PNZ, and the generic product of PNZ.

Figure 3 demonstrates the thermal profiles for the pure powder of PNZ, the brand product of PNZ, and the generic product of PNZ. Slight difference in the endothermic peak temperature corresponding to the melting point between two products of PNZ ( *p > 0.05*) were observed. The generic product had the highest recorded endothermic peak temperature at 164.29°C, as compared to the brand product which exhibited an endothermic peak of 161.74°C. However, the energy absorbed was greater for the brand product at nearly 51.65 J/g and 49.52 J/g for the generic product.

### Thermal gravimetric analysis

**Figure 4.**
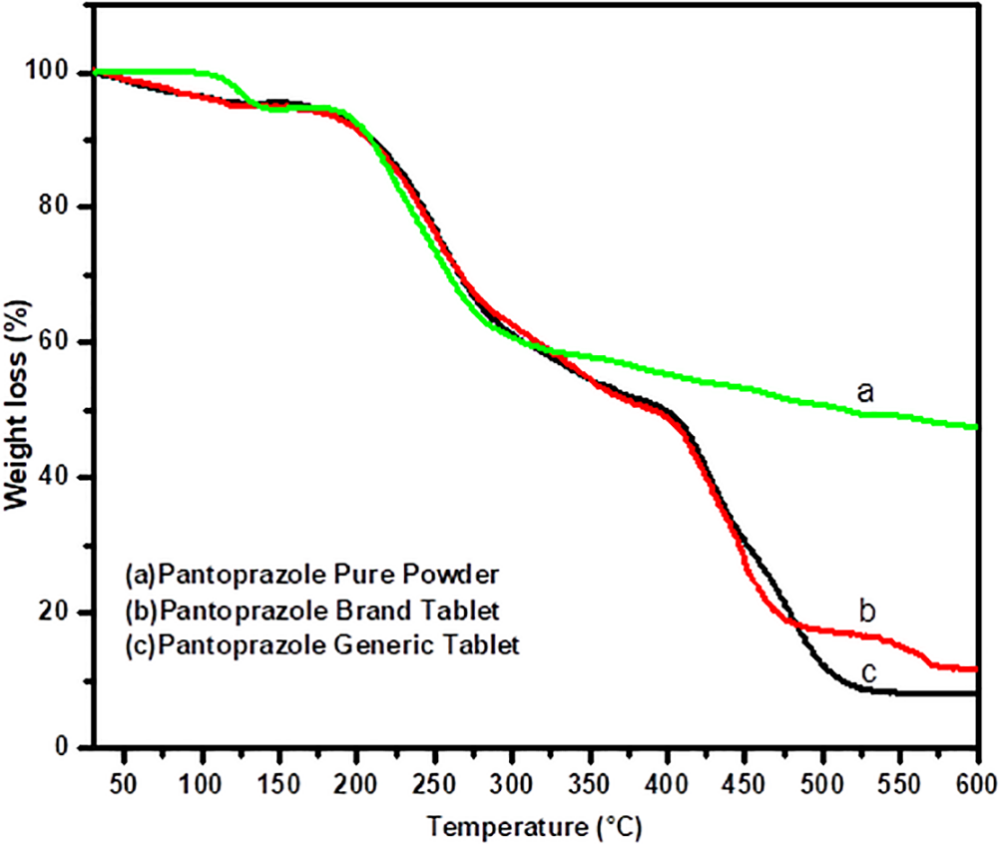
Thermal gravimetric analysis/DTG analytical spectra for the pure powder of PNZ, the brand product of PNZ, and the generic product of PNZ.

Figure 4 represent the thermal profile obtained by TGA for the pure powder of PNZ, The brand product of PNZ and the generic product of PNZ. slight differences were observed among the TGA results of the two samples ( *P > 0.05*). The weight loss for all samples occurred mainly in two stages, with a lower ( *T*_onset_) observed for the generic product compared to the brand product in both stages. In the first stage, the brand product had a ( *T*_onset_) of 140.44°C, and the generic product had a ( *T*_onset_) of 120.00°C. In the second stage, the ( *T*_onset_) values were 379.08°C, and 351.08°C for brand product, and generic product, respectively. The total weight loss for the two samples was slightly higher for the generic product (84.06 %) compared to the brand product (81.58%).

### X-Ray diffraction

**Figure 5.**
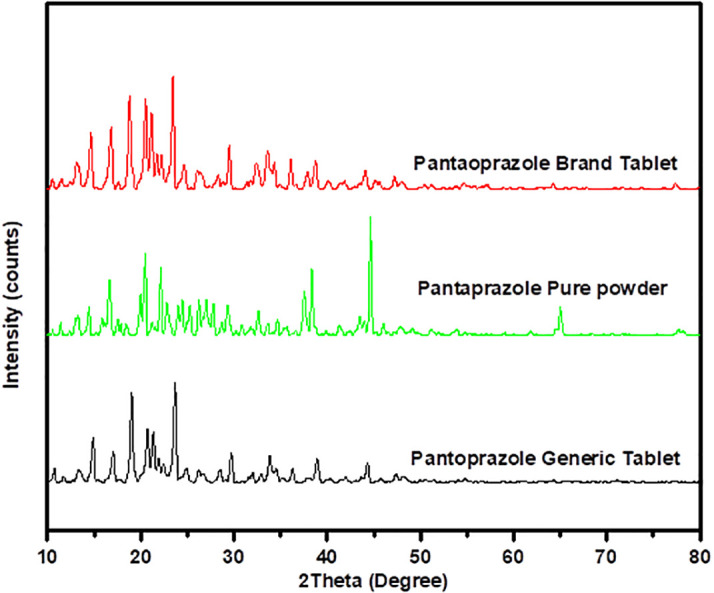
X-ray diffraction analytical spectra for the brand product of PNZ, the pure powder of PNZ, and the generic product of PNZ.

Figure 5 represents the crystalline atomic arrangement of the three samples and shows a clear pattern in their diffraction analysis. Although the peak positions of the three samples were similar, the intensity of the peaks varied between the generic and branded PNZ products. The generic sample exhibited lower peak intensity values compared to the branded sample. The main peaks were observed at 2θ angles of 13°, 14°, 16°, 21°, 39°, and 44°. The differences in peak intensity between the generic and branded products may suggest some variations in the crystalline form of the drug powder in the generic product.

### Scanning electron microscopy

**Figure 6.**
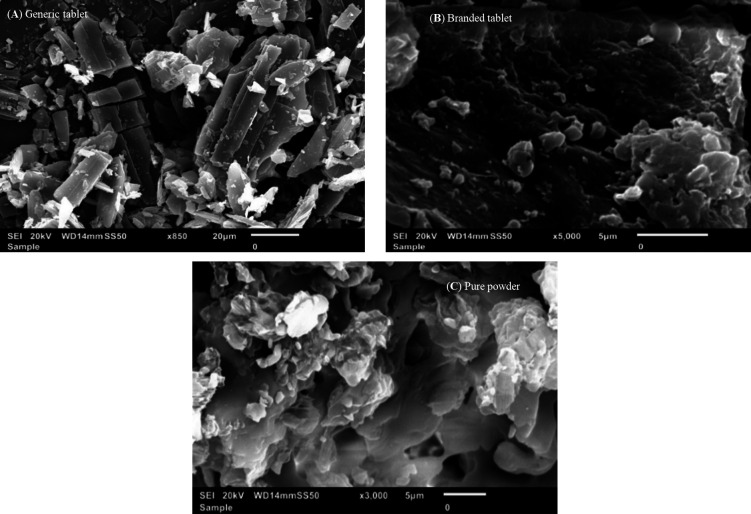
SEM images for (A) generic tablet PNZ, (B) branded tablet of PNZ, and (C) pure powder of PNZ. Caliper indicates 5-20 μm. Images were obtained under x850-5000 - magnifications operating at 20 kV.

The SEM in Figure 6 displays three images: a) the generic product of PNZ, b) the brand product of PNZ, and c) the pure powder of PNZ. These images provide detailed information on the surface morphology, particle size, and shape of the three samples. The pure powder of PNZ and the brand product of PNZ exhibited similar particle sizes, ranging from 10-20 um. In contrast, the generic product of PNZ had a particle size range of 5-10 um. Upon closer examination of the SEM images, it was observed that the pure powder exhibited clear and smooth particle surfaces, whereas the brand product of PNZ and the generic product of PNZ displayed a coarse surface.

### Fourier transforms infrared spectroscopy

**Figure 7.**
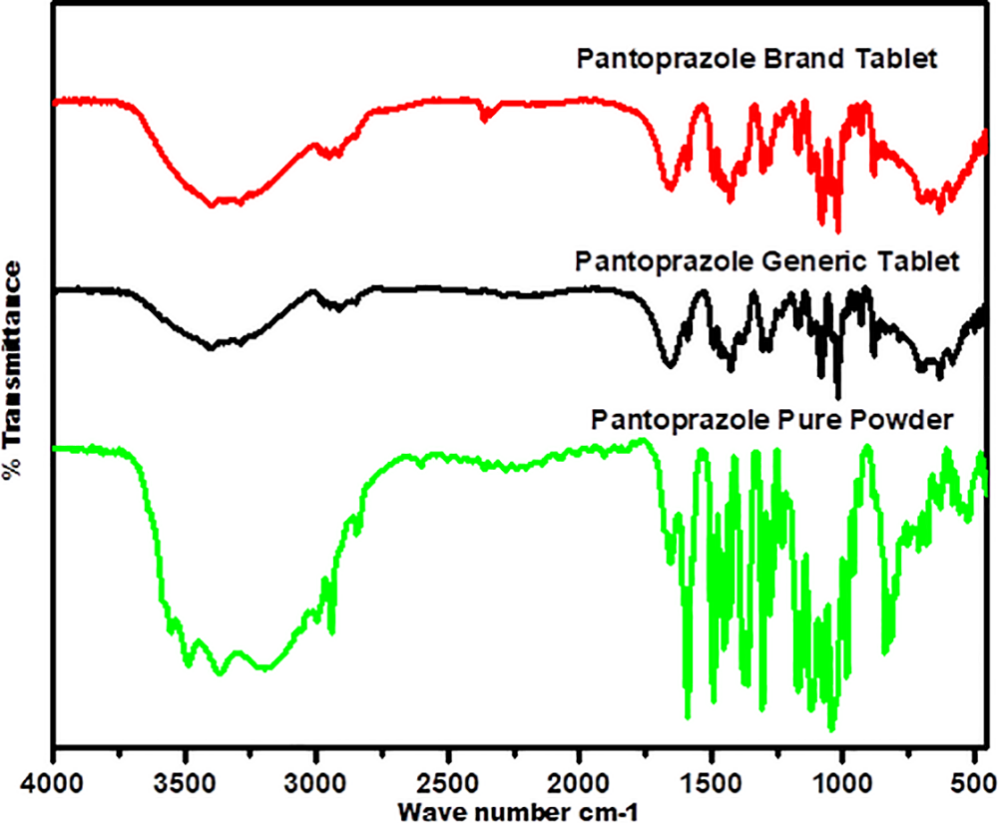
Fourier transform infrared analytical spectra for the generic product of PNZ, the brand product of PNZ, and the pure powder of PNZ.

**Figure 8.**
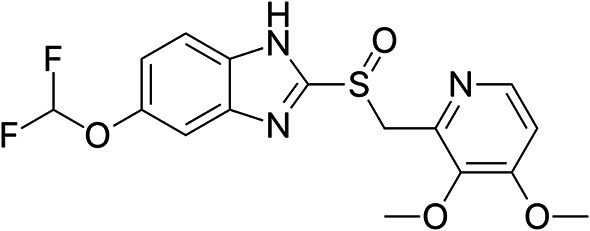
Chemical structure of Pantoprazole.

The FTIR spectra, presented in Figure 7, provide essential qualitative data for the generic product of PNZ by illustrating the bands that correspond to the functional groups of the chemical structures shown in Figure 8. The characteristic bands in the fingerprint regions below 1500 cm ^-1^ demonstrate a similar wave number for the three samples, with less intensity noted for the generic product compared to the other two samples. For example, the C-O stretching vibration is represented by a medium-sharp peak at 1100 cm ^-1^ wave number.

The diagnostic region for the three samples shows almost similar peak positions, with variations in intensity for the generic product of PNZ. The three spectra exhibit the following band positions: N-H bending vibration (secondary amine) at 3400 cm ^-1^, C-H aromatic stretching at 3100 cm ^-1^, carbonyl functional group at 1700 cm ^-1^, and N-H bending vibration at 1589 cm ^-1^ wave number.

### Raman spectroscopy

**Figure 9.**
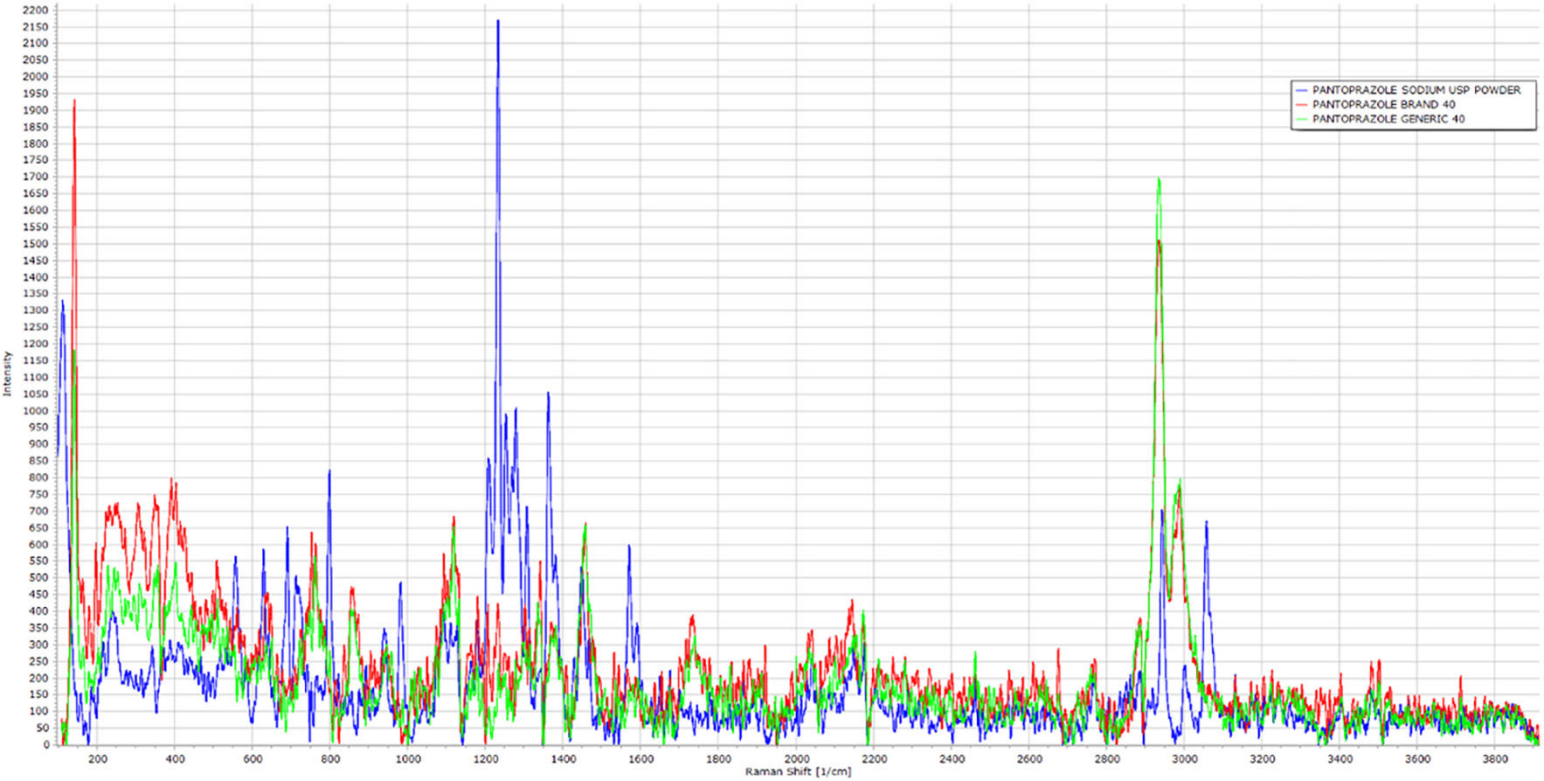
Raman spectroscopy of the pure powder of PNZ, the brand product of PNZ, and the generic product of PNZ.

The spectra profile of the pure powder of PNZ, the brand product of PNZ, and the generic product of PNZ are presented in Figure 9. It was observed that the three spectra had several similar peaks at the same Raman shift, with varying intensities. These peaks mainly corresponded to the functional groups of the PNZ molecule, as shown in Figure 8. The main peaks were demonstrated in Table 1, which included the attributed functional group and intensity for the three different samples.

**Table 1.** Peaks observed in pantoprazole tablets (generic, brand, and PNZ pure powder) used in the study.

	Generic product	Brand product	Pure powder of PNZ	
Raman-shift (1/cm)	*The intensity of the Peak*			Functional Group
241-552	250-550	300-800	100-400	**out-of-plane bending OSCC**
631	300	451	590	**In-plane bending CNC**
945	300	300	350	**N-H wagging**
1092	425	575	425	**stretching CF**
1248	230	420	2175	**stretching OC**
1312	310	430	710	**stretching CC**
3057	175	200	660	**out-of-plane of the C-H bonds in CH3 groups**

## Discussion

To our knowledge, the current study was the first of its kind to highlight the various possible effects of coating material on the protective functionality of enteric coating films for PNZ generic tablets after their recall from the market, using a range of analytical and thermal techniques.

The premature drug release in acidic media for the generic product, compared to the branded product, suggests a weaker resistance of the enteric coating film in the generic tablet. ^37^^,^^54^^,^^55^ Extensive literature indicates that various factors contribute to diminishing the protective functionality of the generic tablet in acidic media. ^37^^,^^56^^–^^58^ Among these factors, the type and amount of excipients used in the enteric coating film have been identified as the primary ones. According to the medication leaflets, both the generic and branded products contain common excipients such as hypromellose, povidone K25, titanium dioxide, yellow iron oxide, propylene glycol, and methacrylic acid ethyl acrylate copolymer (1:1). However, P-80 is present only in the enteric coating film of the branded product.

The presence of P-80 in the enteric coat formulation for the brand product of PNZ is presumed to play a crucial role in preventing premature drug release in acidic conditions. This effect is attributed to the plasticizing properties of P-80, which enhance the integrity of the enteric coating film. ^49^^,^^59^ This finding aligns with several previous research studies that have demonstrated how the addition of P-80 to the enteric coating improves the smoothness and continuity of the film, ultimately preventing cracking in acidic conditions. ^59^^–^^61^ In contrast, the generic product of PNZ does not contain P-80 in its enteric coat formulation, potentially contributing to the premature drug release in acidic media. ^59^ These findings are consistent with previous studies that have shown that the presence of P-80 in enteric coat formulations can enhance the ability of the enteric coat in protecting the tablet from the premature drug release in acidic conditions. ^59^^,^^62^

The DSC thermal analysis profile of the branded and generic products provides valuable information about their respective thermal behaviors. The slight difference in the melting points between the two products indicates the presence of the same drug molecule in both samples, as they fall within the same range for the pure powder of PNZ melting point. ^26^^,^^63^^,^^64^ However, a slight decrease in the area under the curve for the generic product may indicate the presence of some impurities. ^63^^–^^65^ These findings are consistent with several previous research studies that have shown a reverse proportional relationship between the presence of impurities in drug formulations and the corresponding area under the curve in DSC analysis. ^63^^,^^64^ Another factor that could be contributed to the slight variation is the absence of P-80 as a plasticizer in the generic product of PNZ, potentially leading to a higher melting point. ^66^^–^^69^ the presence of a plasticizer in the brand product of PNZ may have slightly lowered its melting point by enhancing flexibility and elasticity. ^66^^–^^69^ These findings align with previous studies that have shown a decrease in the melting point of a formulation upon the addition of a plasticizer. ^68^^–^^70^

The TGA results support the DSC findings. The absence of a plasticizer in the generic tablet may have slightly affected the thermal behavior of the polymer used in the binder, resulting in a higher weight loss during TGA. ^66^^,^^69^ Furthermore, the presence of the plasticizer in the brand tablet may have increased the thermal profile of the polymer in the binder and prevented the onset of weight loss at lower temperatures, leading to a lower weight loss during TGA. ^66^^,^^69^^,^^71^ Overall, these results may demonstrate the possible influence of impurities and P 80 on the thermal behavior of the samples.

The XRD results obtained from the three samples provide valuable information on the crystallite structure, size, and degree of crystallinity of the drug particle. The similarity in the position of the peak for the two products suggests that both the brand and generic products contain the same crystalline structure. ^72^ The observed difference in peak intensity between the pure powder of PNZ and the other two samples might be attributed to the absence of excipients in the pure powder of PNZ. ^69^^,^^71^ This finding was supported by the smooth surface displayed for the pure powder by SEM, whereas the surface of the generic and brand product of PNZ was coarse due to the existence of excipients. ^73^ The higher peak intensity observed for the brand product compared to the generic product may indicate a more ordered crystal structure for the branded product. ^69^^,^^72^ Our results are inconsistent with previous studies, which showed almost similar peak positions for the PNZ drug molecule. ^74^

The FTIR spectra for both the generic and brand products showed similarities in the band positions when compared to the reference pure powder, suggesting that there was no interference between the drug molecule and the excipients used in both products and confirming the presence of the PNZ drug molecule in both. ^60^^,^^75^^,^^76^ However, differences in band transmittance were observed between the generic and brand samples, which could be attributed to the presumed presence of impurities in the generic PNZ product. ^55^^,^^76^^–^^77^ These findings are consistent with previous literature that reported no alteration in the band position for PNZ in the formulation, except for the band transmittance. ^55^ Raman spectra for all three samples showed comparable results to the FTIR, further supporting the concept that the differences observed in the FTIR spectra of the generic and brand products might be due to the presence of impurities. ^55^^,^^76^

## Conclusion

In conclusion, this study highlights the functionality role of the coating materials in the formulation of enteric-coated PNZ generic tablets. It was observed that the variation in the excipients used in the enteric coating of generic products of PNZ affected its protection functionality, leading to premature drug release in acidic media. On the other hand, the brand product showed superior functionality in protecting the drug molecule in acidic media, which might be attributed to the presence of P-80 as a plasticizer and emulsifier in the enteric coating film alongside anionic polymer. Also, the presence of impurities was obvious in the generic product of PNZ. The number of analytical and thermal techniques used in this study provided valuable insights into the differences between the generic and brand products of PNZ. Overall, the findings of this study have significant implications for the development of enteric-coated generic drugs and emphasize the importance of selecting suitable coating materials.

## Data Availability

Figshare: Enteric-Coating Film Effect on the Delayed Drug Release of Pantoprazole Gastro-Resistant Generic Tablets. https://doi.org/10.6084/m9.figshare.23979114.v1. ^78^ This project contains the following underlying data:
-
Data_01_SEM (F1000R).pdf -
Data_4_Xray.pdf -
Data_3_Xray.pdf - Data Prepared for Statistical Analysis of Generic and Brand PNZ_4a.pdf - Dissolution release assessment for PNZ_5a.pdf - Statistical Analysis of Generic and Brand Product Release in Different pH Media_6a.pdf Data are available under the terms of the Creative Commons Attribution 4.0 International license (CC-BY 4.0).
