# Supplementary material for: Enteric-coating film effect on the delayed drug release of pantoprazole gastro-resistant generic tablets
Source: F1000Res. 2023 Oct 12;12:1325. [Version 1] doi: 10.12688/f1000research.140607.1 (PMC11002526; doi:10.12688/f1000research.140607.1)
Supplement: Peer review history

# Reviewer report: Reviewer response for version 1

**DOI:** 10.5256/f1000research.153979.r249484

**Published:** 2024-04-08

**Authors:** Goutam Kumar Jena

**Affiliations:** 1 Roland Institute of Pharmaceutical Sciences, Berhampur, Odisha,, India

The manuscript "Enteric-coating film effect on the delayed drug release of pantoprazole gastro-resistant generic tablets" was found to be interesting. The authors are investigating the effects of enteri-coating effect on the delayed drug release of pantoprazole gastro-resistant generic tablets and compared with the reference standard and concluded that the excipients used in the brand product demonstrated superior functionality in effectively protecting the drug molecule from acidic media through the enteric coating film, as compared to the generic version. The data presentation and results section are good but the discussion section needs to be improved by citing more.

Is the work clearly and accurately presented and does it cite the current literature?

Yes

If applicable, is the statistical analysis and its interpretation appropriate?

Yes

Are all the source data underlying the results available to ensure full reproducibility?

Yes

Is the study design appropriate and is the work technically sound?

Yes

Are the conclusions drawn adequately supported by the results?

Yes

Are sufficient details of methods and analysis provided to allow replication by others?

Yes

Reviewer Expertise:

Nanotechnology, novel drug delivery systems, polymeric nanoparticles and Nano structured lipid carriers

I confirm that I have read this submission and believe that I have an appropriate level of expertise to confirm that it is of an acceptable scientific standard.

# Reviewer report: Reviewer response for version 1

**DOI:** 10.5256/f1000research.153979.r249483

**Published:** 2024-03-20

**Authors:** Jiyauddin Khan

**Affiliations:** 1 School of Pharmacy, Management and Science University, Shah Alam, Selangor, Malaysia

**REVIEWER COMMENTS: A & B**

**(A). **The manuscript entitled **“** **ENTERIC-COATING FILM EFFECT ON THE DELAYED DRUG RELEASE OF PANTOPRAZOLE GASTRO-RESISTANT GENERIC TABLETS** **” **was reviewed prudently, and it was felt that this type of research work contributes to the pharmaceutical science in terms of developing several solid dosage forms.

 This manuscript is well written scientifically & grammatically. On a scientific ground, the idea of the manuscript is novel and significant. The paper contains new information that is adequate to justify publication but suggests some minor revisions to the manuscript. Overall, the manuscript is good and well-written.

**(B).** 

 A few minor corrections are suggested, and comments to the authors are listed below.

**Scientific Comment & Suggestions:**

**1. The introduction** **part** is too general; it is required to write up some more if possible. I suggest elaborating on the strength of the concern area will be a more  comprehensive approach than describing the general contents.

**2. The literature review** given in the manuscript is substantial, with considerable planning, methodical searches, and attention to detail. The information covered in the process has provided valuable input for the research.

**3. The research methodology** in the manuscript has covered all study design, data, & statistical analysis, as well as ethical considerations. 

**4. Results and discussions** are presented and comply with the manuscript, which required fitting with the aims and objectives of the research work. The discussion of research is done extensively and is appropriately compared with other research findings, etc.

**5. The research findings** of the current research works are concluded properly, if possible, can be concluded with appropriate **recommendations,** and **further suggestions** etc.

**6. I verified that all the references** given are cited in this manuscript. However, the formatting of the references requires multiple citation formats (APA, Harvard, MLA, and Chicago manual style). The date of reference should be mentioned, and the internet citations are changed periodically. I request that you please follow only one citation format and apply it to all. 

**7. The figure and table** are properly placed in the correct location according to their descriptions.

**General Comments:**

 I do accept that as we all are non-English speakers the language proficiency will always be a barrier to maintain the standards. Therefore, I suggest the candidate do thorough language editing for this manuscript.

Is the work clearly and accurately presented and does it cite the current literature?

Yes

If applicable, is the statistical analysis and its interpretation appropriate?

Yes

Are all the source data underlying the results available to ensure full reproducibility?

Yes

Is the study design appropriate and is the work technically sound?

Yes

Are the conclusions drawn adequately supported by the results?

Yes

Are sufficient details of methods and analysis provided to allow replication by others?

Yes

Reviewer Expertise:

Extremely interested in Research Oriented Works of Pharmaceutical Technology (Drug Development, Dosages form Design, Drug Delivery and Novel Drug Delivery Systems) related to Controlled-release/ Sustained-release Matrix Formulations etc. Beside these interests, also I am highly interested in Clinical Trials / Bio-equivalence Studies, Halal Pharmaceuticals & Cosmeceuticals etc.

I confirm that I have read this submission and believe that I have an appropriate level of expertise to confirm that it is of an acceptable scientific standard.
